# Author Correction: Toddlers strategically adapt their information search

**DOI:** 10.1038/s41467-024-51561-w

**Published:** 2024-08-21

**Authors:** Francesco Poli, Yi-Lin Li, Pravallika Naidu, Rogier B. Mars, Sabine Hunnius, Azzurra Ruggeri

**Affiliations:** 1 Donders Institute for Brain, Cognition and Behaviour, Radboud University, Nijmegen, Netherlands; 2 MRC Cognition and Brain Sciences Unit, University of Cambridge, Cambridge, UK; 3 Wellcome Centre for Integrative Neuroimaging, Centre for Functional MRI of the Brain (FMRIB), Nuffield Department of Clinical Neurosciences, John Radcliffe Hospital, University of Oxford, Oxford, UK; 4 Max Planck Research Group iSearch, Max Planck Institute for Human Development, Berlin, Germany; 5 School of Social Sciences and Technology, Department of Education, Technical University Munich, Munich, Germany; 6 Department of Cognitive Science, Central European University, Vienna, Austria

**Keywords:** Human behaviour, Problem solving

Correction to: *Nature Communications* 10.1038/s41467-024-48855-4, published online 10 July 2024

In this article the affiliation ‘Max Planck Research Group iSearch, Max Planck Institute for Human Development, Berlin, Germany’ was incorrectly given for Rogier Mars but should have been removed. In addition, the affiliation ‘Wellcome Centre for Integrative Neuroimaging, Centre for Functional MRI of the Brain (FMRIB), Nuffield Department of Clinical Neurosciences, John Radcliffe Hospital, University of Oxford, Oxford, UK’ was incorrectly given for Azzurra Ruggeri but should have been ‘Max Planck Research Group iSearch, Max Planck Institute for Human Development, Berlin, Germany’. The original article has been corrected.

