# Supplementary material for: Toddlers strategically adapt their information search
Source: Nat Commun. 2024 Jul 10;15:5780. doi: 10.1038/s41467-024-48855-4 (PMC11237003; doi:10.1038/s41467-024-48855-4)
Supplement: Supplementary file 2 — Description of Additional Supplementary Files [file 41467_2024_48855_MOESM2_ESM.pdf]

Description of Additional Supplementary Files

File Name: Supplementary Movie 1
Description: Example of the task recorded from an adult participant. Treasure chest images with copyright from Babysofja via Creative Market and animal images freely available from Prora via Pixabay. Audio files were downloaded from ZapSplat.
