# Supplementary material for: Toddlers strategically adapt their information search
Source: Nat Commun. 2024 Jul 10;15:5780. doi: 10.1038/s41467-024-48855-4 (PMC11237003; doi:10.1038/s41467-024-48855-4)
Supplement: Supplementary file 4 — Reporting Summary [file 41467_2024_48855_MOESM4_ESM.pdf]

nature portfolio

Corresponding author(s): Francesco Poli

Last updated by author(s): Apr 2, 2024

Reporting Summary

Nature Portfolio wishes to improve the reproducibility of the work that we publish. This form provides structure for consistency and transparency in reporting. For further information on Nature Portfolio policies, see our Editorial Policies and the Editorial Policy Checklist.

Statistics

For all statistical analyses, confirm that the following items are present in the figure legend, table legend, main text, or Methods section.

n/a	Confirmed	
☐	☒	The exact sample size (*n*) for each experimental group/condition, given as a discrete number and unit of measurement
☐	☒	A statement on whether measurements were taken from distinct samples or whether the same sample was measured repeatedly
☐	☒	The statistical test(s) used AND whether they are one- or two-sided<br>*Only common tests should be described solely by name; describe more complex techniques in the Methods section.*
☐	☒	A description of all covariates tested
☐	☒	A description of any assumptions or corrections, such as tests of normality and adjustment for multiple comparisons
☐	☒	A full description of the statistical parameters including central tendency (e.g. means) or other basic estimates (e.g. regression coefficient) AND variation (e.g. standard deviation) or associated estimates of uncertainty (e.g. confidence intervals)
☐	☒	For null hypothesis testing, the test statistic (e.g. *F*, *t*, *r*) with confidence intervals, effect sizes, degrees of freedom and *P* value noted<br>*Give P values as exact values whenever suitable.*
☒	☐	For Bayesian analysis, information on the choice of priors and Markov chain Monte Carlo settings
☒	☐	For hierarchical and complex designs, identification of the appropriate level for tests and full reporting of outcomes
☐	☒	Estimates of effect sizes (e.g. Cohen's *d*, Pearson's *r*), indicating how they were calculated

Our web collection on statistics for biologists contains articles on many of the points above.

Software and code

Policy information about availability of computer code

Data collection	Data was collected with Python 3.6 using custom scripts.
Data analysis	Data was analyzed with Python 3.6 with the hmmlearn package, and in R 4.1 using the mgcv and emmeans packages.<br>Code availability<br>Code for the task implementation, statistical analyses, and computational modelling can be found on OSF: https://osf.io/rfx5u/.

For manuscripts utilizing custom algorithms or software that are central to the research but not yet described in published literature, software must be made available to editors and reviewers. We strongly encourage code deposition in a community repository (e.g. GitHub). See the Nature Portfolio guidelines for submitting code & software for further information.

Data

Policy information about availability of data

All manuscripts must include a data availability statement. This statement should provide the following information, where applicable:

- Accession codes, unique identifiers, or web links for publicly available datasets
- A description of any restrictions on data availability
- For clinical datasets or third party data, please ensure that the statement adheres to our policy

Data availability
Data can be found on OSF: https://osf.io/rfx5u/.

Research involving human participants, their data, or biological material

Policy information about studies with human participants or human data. See also policy information about sex, gender (identity/presentation), and sexual orientation and race, ethnicity and racism.

Reporting on sex and gender	Adult participants were asked to share their gender if they were comfortable in doing so. Caregivers of infant participants were asked for their infant's sex at birth. All the procedures followed the Ethical Guidelines of the Max Planck Institute and were approved by the Ethics Committee. Differences across genders in adults, and across sexes in infants were not analysed, because we did not expect differences across gender or sex.
Reporting on race, ethnicity, or other socially relevant groupings	The population characteristics are reported in the main manuscript as follows: Forty-two adults (M = 37.02 years, SD = 16.77, range 18-73 years, F = 24, M = 18) with normal to corrected vision and no mobility disorders as prerequisites, as well as 60 toddlers (Mean = 27.36 months, SD = 0.37, range 17.8-35.8 months, F = 35, M = 25) were recruited.
Population characteristics	Adult participants were asked to share their gender if they were comfortable in doing so. Caregivers of infant participants were asked for their infant's sex at birth. All the procedures followed the Ethical Guidelines of the Max Planck Institute and were approved by the Ethics Committee. Differences across genders in adults, and across sexes in infants were not analysed, because we did not expect differences across gender or sex.
Recruitment	Participants were recruited from the online database of the Max Planck Institute for Human Development, Berlin, Germany. Regarding self-election biases, participants joining from the online registration system are unlikely to be elderly. Given that we were not intersted in this population, this self-selection bias has no effect on the results.
Ethics oversight	The study was approved by the Ethics Committee of the Max Planck institute for Human Development, Berlin, Germany. Informed consent was obtained from adults and from children's legal guardians prior to participation.

Note that full information on the approval of the study protocol must also be provided in the manuscript.

Field-specific reporting

Please select the one below that is the best fit for your research. If you are not sure, read the appropriate sections before making your selection.

☐ Life sciences ☒ Behavioural & social sciences ☐ Ecological, evolutionary & environmental sciences

For a reference copy of the document with all sections, see nature.com/documents/nr-reporting-summary-flat.pdf

Behavioural & social sciences study design

All studies must disclose on these points even when the disclosure is negative.

Study description	The study was a quantitative experimental study that focused on two samples of participants (toddlers and adults).
Research sample	The research sample consisted of adults and toddlers living in Berlin and neighbouring areas. Participation was open to anyone, via registration to the online database of the Max Planck institute for Human Development, Berlin, Germany. Participants were recruited from the online database of the Max Planck Institute for Human Development, Berlin, Germany. Forty-two adults (M = 37.02 years, SD = 16.77, range = 18 - 73 years, F = 24, M = 18) with normal to corrected vision and no mobility disorders as prerequisites, as well as 60 toddlers (Mean = 27.36 months, SD = 0.37, range = 17.8 – 35.8 months, F = 35, M = 25) were recruited. To compute the sample size, we simulated synthetic data using information from pilot data (N=5) and data from a study with a similar design and population (N=37), as well as theoretical constraints (code is available at https://osf.io/rfx5u/).We simulated the expected results 1000 times and identified that power above 80% was expected to be reached with a sample of toddlers >= 59 (Mean power = 86.60%, CI = [84,33, 88.65]) and a sample of adults >= 28 (Mean power = 85.00%, CI = [82.63, 87.16]) due to reduced noise in adult data.
Sampling strategy	Participants were divided into the two pseudo-randomization groups based on their participation numbers. Odd numbers were assigned to group A, even numbers were assigned to group B. The experimenters testing the participants were unaware of this randomization method. See Research Sample above for information regarding sample size.
Data collection	Data was collected with a Tobii Eye Tracker. The data collection sessions were video recorded. Testers were bling to the experimental conditions and hypotheses. When infants were tested, the caregiver was always present in addition to the tester.
Timing	Data collection started on February 23, 2022, and ended on May 18, 2022.
Data exclusions	No data was excluded from the analyses
Non-participation	No participants dropped out of the study
Randomization	Participants were allocated to different pseudorandomizations of the same stimuli depending on the participant number (even vs odd). The pseudorandomizations were added as random effects in the analyses.

Reporting for specific materials, systems and methods

We require information from authors about some types of materials, experimental systems and methods used in many studies. Here, indicate whether each material, system or method listed is relevant to your study. If you are not sure if a list item applies to your research, read the appropriate section before selecting a response.

Materials & experimental systems

n/a	Involved in the study
☒	☐ Antibodies
☒	☐ Eukaryotic cell lines
☒	☐ Palaeontology and archaeology
☒	☐ Animals and other organisms
☒	☐ Clinical data
☒	☐ Dual use research of concern
☒	☐ Plants

Methods

n/a	Involved in the study
☒	☐ ChIP-seq
☒	☐ Flow cytometry
☒	☐ MRI-based neuroimaging

Plants

Seed stocks	*Report on the source of all seed stocks or other plant material used. If applicable, state the seed stock centre and catalogue number. If plant specimens were collected from the field, describe the collection location, date and sampling procedures.*
Novel plant genotypes	*Describe the methods by which all novel plant genotypes were produced. This includes those generated by transgenic approaches, gene editing, chemical/radiation-based mutagenesis and hybridization. For transgenic lines, describe the transformation method, the number of independent lines analyzed and the generation upon which experiments were performed. For gene-edited lines, describe the editor used, the endogenous sequence targeted for editing, the targeting guide RNA sequence (if applicable) and how the editor was applied.*
Authentication	*Describe any authentication procedures for each seed stock used or novel genotype generated. Describe any experiments used to assess the effect of a mutation and, where applicable, how potential secondary effects (e.g. second site T-DNA insertions, mosiacism, off-target gene editing) were examined.*
